# Cervical spine involvement in pediatric mucopolysaccharidosis patients: Clinical features, early diagnosis, and surgical management

**DOI:** 10.3389/fsurg.2022.1059567

**Published:** 2023-01-06

**Authors:** Hai-Tao Liu, Jia Song, Fu-Chao Zhou, Zhi-Hui Liang, Qiu-Qi Zhang, Yue-Hui Zhang, Jiang Shao

**Affiliations:** Spine Center, Xin Hua Hospital, Affiliated to Shanghai Jiao Tong University School of Medicine, Shanghai, China

**Keywords:** mucopolysaccharidoses, cervical spine, surgical managements, occipitocervical fusion, atlantoaxial fusion

## Abstract

Mucopolysaccharidosis (MPS) is a progressive genetic disease that causes a deficiency in lysosomal enzymes, which play an important role in the degradation pathway of glycosaminoglycans. As a result of enzyme defects, mucopolysaccharides cannot be metabolized and thus accumulate. The cervical spine is one of the most commonly involved sites; thus, prompt surgical management before the onset of severe neurological deterioration is critical. However, because of the rarity of the disease, there is no standard treatment. In this review, we characterize the cervical spinal involvement in pediatric patients with MPS, describe the useful imaging technologies for diagnosis, and provide screening procedure for children with MPS. Surgical managements, including indications, surgical methods, possible difficulties, and solutions, are reviewed in detail.

## Introduction

Mucopolysaccharidoses (MPSs) are a group of hereditary lysosomal storage diseases caused by a deficiency of an enzyme that plays an important role in the degradation pathway of glycosaminoglycans (GAGs). Except for Hunter syndrome (MPS type II), which has an X-linked recessive inheritance, all MPSs have autosomal recessive inheritance (1). The lack of enzymes leads to the storage of corresponding GAGs, which are considered to be the primary and direct cause of MPS (1). MPSs can be classified into seven types according to the type of the enzyme deficiency, and some of them can be further divided into subcategories (Table 1). In total, there are 12 different types and subtypes of MPSs, including the recently described mucopolysaccharidosis-plus syndrome (MPSPS). In MPSPS, heparan sulfate is stored, but there is no enzyme deficiency (2).

**Table 1:** Different types of MPS.

Types	Deficient enzyme	Accumulated GAGs	Thoracolumbar kyphosis	Odontoid dysplasia
MPS I (Hurler syndrome)	*α*-L-iduronidase	DS, HS	+	+
MPS II* (Hunter syndrome)	Iduronate-2-sulfatase	DS, HS	+	+
MPS III (Sanfilippo syndrome)	Subtype A: Heparan-N-sulfatase	HS		
	Subtype B: α-N-acetyglucosaminidase			
	Subtype C: α-glucosaminidase-acetyltransferase			
	Subtype D: N-acetylglucosamin-6-sulfatase			
MPS IV (Morquio syndrome)	Subtype A^a^: N-acetylglucosamin-6-sulfatase	Subtype A: KS, C6S	+	+
	Subtype B: *β*-galactosidase	Subtype B: KS		
MPS VI (Maroteaus-Lamy syndrome)	N-acetylgalactosamine-4-sulfatse	DS, C4S	+	+
MPS VII (Sly syndrome)	β-glucuronidase	DS, HS, C4S, C6S	+	
MPS IX (Natowicz syndrome)	Hyaluronidase I	H		
MPSPS	Not found	HS	+	

MPSPS, Mucopolysaccharidosis-plus syndrome; DS, dermatan sulfate; HS, heparan sulfate; KS, keratan sulfate; C6S, chondroitin-6-sulfate; C4S, chondroitin-4-sulfate; H, Hyaluronan.

^a^ Main type of MPS that influences the cervical spine.

GAGs accumulating in the cells affect cellular processes such as cell adhesion and signaling, causing multiorgan and severe symptoms such as coarse facial features, cognitive retardation, hepatosplenomegaly, hernias, kyphoscoliosis, and corneal clouding (1). Skeletal involvements are the most common manifestations and have been reported in all subtypes of MPS except in MPS type IX. Most MPS types, especially types I and IV, have cervical spine involvement, presenting as an absence of the odontoid process, atlantoaxial dislocation, spinal canal stenosis and compression, and others. In the study of Remondino et al. (3), 43 of 52 patients with MPS had cervical diseases, and odontoid hypoplasia, along with subsequent atlantoaxial instability, was frequently observed in those patients. If it is not addressed, it will develop into myelopathy, which can be life-threatening (4).

Early diagnosis allows early intervention, thus improving the chances of a better outcome. At present, the diagnosis of MPS is relatively mature, including blood and urinary GAG tests, enzyme assays, and genetic tests (1). The development of imaging technology is of great importance in the diagnosis and preoperative evaluation of cervical involvement. Furthermore, close collaboration between clinicians and radiologists is essential (5). However, for those young patients, how to conduct screening and evaluation is still a problem to be solved.

Treatment of the etiology and the corresponding symptoms of MPS in the spine should be comprehensive, involving multiple disciplines. Etiological treatment mainly refers to the disease-specific treatment of MPS, including enzyme replacement therapy and hematopoietic stem cell transplantation, as well as new approaches, such as gene therapy, substrate reduction therapy, chaperone therapy, and combinations of these strategies. However, as MPS is a progressive disease and the lifespan of patients with MPS has increased with improvements in the medical treatment of MPS (6), and considering that the existing strategies cannot correct the pathological damage that has occurred, especially for bone damage, surgical intervention before the onset of serious consequences has become a last-resort, albeit a very effective, strategy.

Generally, the biggest threat to children with MPS may be damage to the heart and respiratory system. However, with the development of diagnostic and treatment technologies, MPS can be diagnosed and treated effectively at an early stage, which means that cardiopulmonary damage can be effectively controlled and the life expectancy can be prolonged. As existing treatments for bones and cognitive damage have limited effectiveness and cannot effectively prevent the progress of bone damage, and coupled with the prevalence of cervical involvement, early diagnosis of cervical involvement and prompt surgical intervention have become more important.

To improve outcomes in patients with MPS, this review characterizes the cervical spinal involvement and related factors, briefly describes the imaging tools for early diagnosis, provides a screening procedure for children with MPS, and discusses the possible surgical interventions for pediatric patients with MPS with cervical involvements.

## Spinal involvements of MPS

Skeletal manifestations have been reported in all subtypes of MPS except in MPS type IX, and spinal manifestations are particularly common in the spectrum of skeletal disease in these patients (4). The degree of severity varies among different subtypes, which may be related to both the type and quantity of the accumulated GAG fragments. Even if they are of the same type, the severity also varies, which is considered to be the result of differences in the exact mutation site among patients (4).

Spinal cord compression in the cranial segment caused by MPS is the most important condition and usually needs surgical intervention. This condition can directly cause neurological damage, which may present as cervical pain, unsteady gait, frequent falls, progressive impairment of autonomous ambulation, and/or acute tetraplegia after even minor trauma (7). The main direct causes of spinal cord compression include atlantoaxial subluxation, thickening of the dura, and hypertrophy of the ligamentum flavum.

Atlantoaxial subluxation is often the direct cause of cervical spinal cord compression, which can occur anteriorly, posteriorly, vertically, laterally, or in combinations (5). A pincer-like effect is caused by atlantoaxial subluxation between the posterior arch of the atlas and the axis, indenting the dorsal aspect of the spinal cord and causing further compression of the cord (5). For patients with MPS, the absence of the odontoid process is common and may be one of the main causes of atlantoaxial joint instability. Cervical spinal cord compression without dens hypoplasia is unusual, and only one such case has been reported (8). An unstable atlantoaxial joint further develops into atlantoaxial subluxation, which leads to spinal cord compression. Combined with relaxed ligaments, which is also common in patients with MPS, the risk of atlantoaxial subluxation greatly increases (4). This unstable situation poses a hidden, yet grave, danger and requires surgical intervention as soon as possible.

In addition, the thickened dura and hypertrophied ligamentum flavum are also important causes of compression of the spinal cord, which may be the result of the accumulation of GAGs. Different from C1-C2 instability, there is no ideal surgical method to address the problem of dura hypertrophy owing to its diffuse nature and important role in accommodating cerebrospinal fluid (CSF) (4).

## Imaging techniques for early diagnosis

Skeletal and spinal manifestations are important clinical manifestations of MPS and may even be the first sign of MPS; for example, kyphosis is often the first sign of MPS type IV in early childhood despite a healthy appearance at birth (1, 9). Therefore, radiographic findings are essential for the diagnosis of MPS, especially for spinal diseases. Atlantoaxial dislocation and kyphosis are the most common spinal manifestations in MPS, which can be detected on roentgenography and three-dimensional computed tomography (3D CT) reconstruction. The presence of characteristic vertebral anomalies, such as a “beaked” vertebral body, provide clues for diagnosing MPS (9). Lateral plain-film flexion-extension studies are typically used to detect atlantoaxial instability, subluxation, and dislocation (5, 10), but their usefulness is limited in some patients with basilar invagination or enlarged mastoid processes, as the C1-C2 level is not clearly visible.

**Figure 1:**
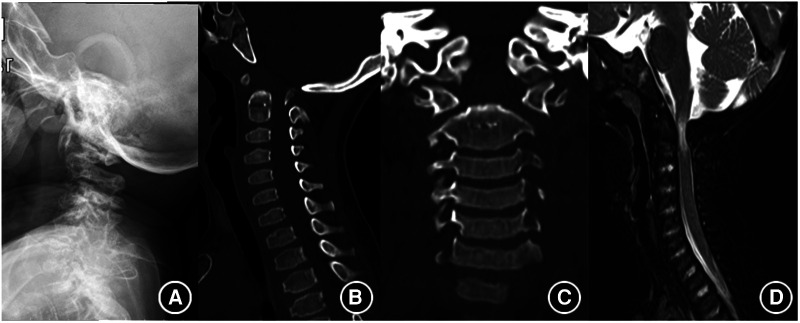
Typical imaging findings of MPS. (**A**) Shows atlantoaxial dislocation on roentgenography. (**B,C**) Shows the absence of odontoid process. (**D**) Shows the compression of the spinal cord.

Magnetic resonance imaging (MRI) can visualize the spinal cord and spinal canal; thus, it is the most useful technology for determining whether there is a compression of the spinal cord and for monitoring nervous complication of MPS spinal involvement, which, in turn, helps us determine whether surgical intervention is needed (5). Spinal stenosis with loss of CSF flow on MRI suggests spinal cord compression. It is worth noting that the nervous deficit is not as severe as that suggested by imaging; thus, comprehensive consideration is needed to make an accurate judgment (Figure 1).

The development of imaging technology has greatly helped in the diagnosis, but some problems remain. Although CT can clearly show the bony structure, it involves exposure to a large dose of radiation; thus, it should be carried out cautiously in young children. Meanwhile, most young patients find it difficult to tolerate the long examination time and loud noise during MRI examination. The usual practice is to sedate the child, but sedatives have substantial risks that cannot be ignored. To reduce the risk for young patients, we should try to minimize examination times. Anesthetization should be carried out by experienced anesthesiologists.

**Figure 2:**
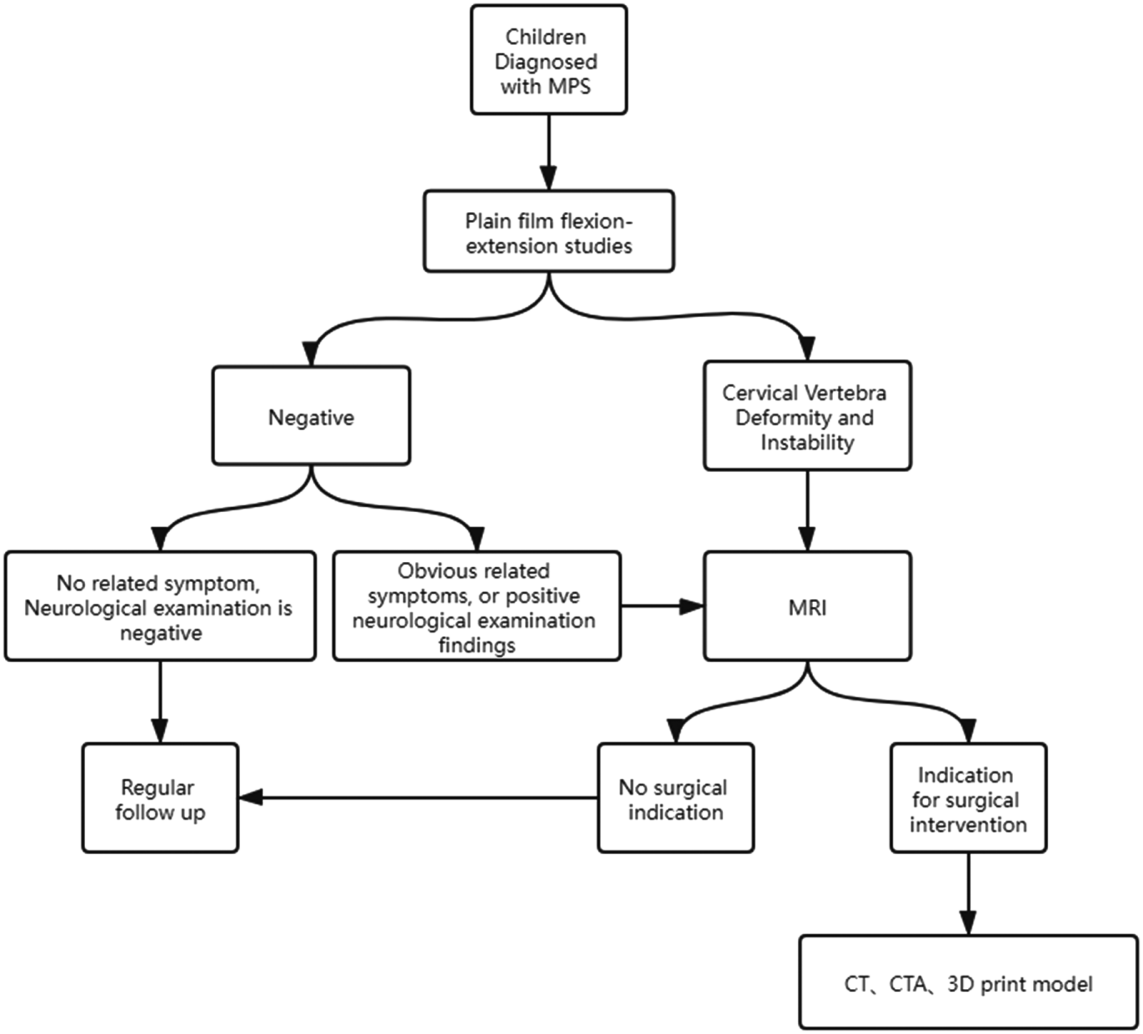
Screening procedure for children diagnosed with MPS.

Owing to the severity and inevitability of cervical spinal cord involvement in patients with MPS, we believe that the following screening procedures are necessary for children with MPS with or without obvious related clinical manifestations, as some patients with MPS (especially type IV) may appear healthy at birth and spinal abnormalities only appear in early childhood (Figure 2). First, cervical anterior, lateral, and dynamic lateral flexion-extension radiographs should be performed routinely to detect cervical deformity and joint instability in the early stage. In view of the potential damage of CT and MRI to young patients, if a child has no positive radiographic findings or related symptoms and a negative neurological examination, further radiological examination is not necessary for the time being, but regular follow-up is needed. If there are positive findings, related clinical symptoms, or neurological signs, furtherMRI examinations are recommended to accurately determine the existence and severity of spinal canal stenosis, and spinal cord compression and to determine whether there are surgical indications. As MRI, including active dynamic flexion and extension scans, is the most useful technique to detect spinal cord compression, it was recommended to be carried out every year (11). CT was suggested to be reserved for children who was considered for surgical procedure (7, 11). If there are surgical indications, CT angiography is also recommended before the operation plan is made to determine the location of blood vessels at the surgical site, which is especially important for patients with developmental malformations. We believe that this screening procedures is helpful for the early diagnosis of cervical lesions in children with MPS, and can provide some reference for spinal surgeons.

## Management of spinal involvements

Because of the low incidence of MPS, no unified consensus on surgical indications exists, and applicable literature is scarce (related studies are summarized in Table 2). Although some studies believe that surgical interventions do not halt neurological progression in most preoperatively clinically symptomatic patients, surgical intervention at an early age is still advocated because early intervention before clinical symptoms is of vital importance for long-term neurological preservation (10). With the development of imaging technologies, attempts on a more nuanced approach rely on a combination of clinical examination and radiology, particularly on MRI (5, 11). Unfortunately, there seem to be no convincing research results so far. Based on our analysis and summary of existing literature, combined with our clinical experience, we recommend the following indications for surgical intervention: increasing cervical cord compression in MRI, with or without notable myelopathy; evidence of instability on cervical dynamic lateral flexion and extension radiography; progressive clinical neurological signs with seemingly non-progressive radiological changes (10). For children with definite absence of odontoid process, surgical intervention should also be carried out promptly, because this condition has the potential danger of atlantoaxial dislocation and further nerve damage.

**Table 2:** Summary of related researches on surgical treatment of MPS.

Author & Year	Number of patients	Mean age	MPS type (Type, N)	Surgical Type (extent)	Fixation	Graft	Complications	Follow-up Time and Result
Marco Crostelli, et al, 2019 (9)	1	4Y6M	I	Posterior occiput-cervical decompression and occipital cervical stabilization (C0-C1)	Rods, occipital screws, and cervical hooks	NR	Soft tissues swelling by protruding instrumentation	6M: Regained partial use of upper extremities 11Y: Complete fusion, with instrumentation completely embedded in fusion mass; full stable neurologic status
odrigo G. Remondino, et al, 2019 (3)	21	8Y	I, II, III, IV, V	Cervical decompression without instrumented fusion^a^ (*n* = 7) Cervical decompression with instrumented fusion (*n* = 6) Instrumented fusion (C1-C2, *n* = 6) Laminectomies without instrumented fusion (*n* = 3)	Microplate and fiber wire (*n* = 2); Robs, screws and hooks (details NR)	Mixture of local and autologous bone graft	Proximal junctional kyphosis (*n* = 6) Cervical pseudarthrosis (*n* = 5) Neurologic impairment that lead to revision surgery (*n* = 6) Wound infection (*n* = 4)	4M: Neurologic improvement (*n* = 6) Unchanged neurologic status (*n* = 9) Deterioration of neurologic status (*n* = 6)
Harald Krenzlin, et al, 2017 (12)	15	14.9 ± 8.2Y	I (*n* = 5) IVA (*n* = 5) VI (*n* = 5)	Stand-alone decompression	/	/	Restenosis (*n* = 7) Re-opreation (*n* = 9) Rate of respiratory-related complication 13.4%	6 ± 5 Y: No patient developed signs of C0-C1-C2 instability or progressive myelopathy.
A.Broomfield, et al, 2018 (10)	26	6.1 Y (1.45 to 15.24)	IVA	Posterior occipito-cervical fixation and fusion using both instrumentation and bone graft, 7 cases require decompression	Wire fixation	Autologous bone graft from alvarial donor site	Reoperation (*n* = 7): Failure of graft fusion at initial operation with subsequent clinical neurological progression (*n* = 2) Radiological signs of worsening stenosis (*n* = 2); Head fixation-pin-related complications (*n* = 3)	84M (7 to 191): Of the 14 preoperatively clinically asymptomatic patients: Neurologically stable (*n* = 14) Of the 10 preoperatively clinically symptomatic patients: Initially improved (*n* = 2) Neurological deterioration in perioperative period (*n* = 2); Improved post operation (*n* = 1); Improved and then declined further (*n* = 1); Remained stable (*n* = 1) Continued to deteriorate (*n* = 5)
Michael C. Ain, et al, 2006 (38)	7	12Y (3 to 46)	IVA	Cervical arthrodesis C1-2 (*n* = 4) C0-4 (*n* = 1) C1-4 (*n* = 1) C0-2 (*n* = 1)	Wire fixation; robs; screws; (details NR)	Iliac crest	None	5.2M Osseous union:7 Neurologic improvement: 4 Unresolved neurologic manifestation:1 Normal neurologic function:2
John K. Houten, et al, 2011 (37)	1	17Y	IVA	Posterior fusion (C0-C4)	Instrumentation with screw fixation in the occiput, C3 and C4 lateral masses and C2 pars	Allograft ribs wrapped with bmp-2 sponges	None	The patient's gait and hand strength improved, and she remains neurologically stable at the 5-year follow-up. A CT scan 10 months following surgery documented maintenance of anatomic alignment and a solid fusion.
Klane K. White, et al, 2009 (36)	1	5Y	IVA	Posterior decompression of C1 and fusion from the occiput to C2 (C0-C2)	Wire fixation	Gallie bone graft	None	26 Y: He slowly regained full neurological function and walking ability. Radiographic examination demonstrated propagation of the fusion down to C3 at the time of the most recent follow-up.
Petr Vanek, et al, 2015 (39)	4	12Y (10 to 14)	IVA	Decompression and instrumented fusion (C0–C2)	2 occiput rods; 1 occipital plate; 2 C2 bilateral laminar screws	A mixture of local bone together with bone graft substitute	None	2Y: Neurologically stable; The control CT scan revealed a stable position of the treated segments, but solid bony fusion was not registered in any patient.
EunJi Moon, et al, 2020 (14)	1	3Y	IVA	Atlantoaxial fixation with C1 and C2 pedicle screw insertion and decompression (C1-C2)	Pedicle screw at the left C2 vertebra; translaminar screw at the right C2 lamina; C1 pedicle screw; rod connection	Local bone chips harvested from the C2 spinous process into both facet joint spaces	None	8M: Postoperative stable C1-C2 fixation. Able to stand up and walk with minimal assistance in a short time; 8 months postoperatively, the patient's quadriparesis was improved to nearly grade V, with the ability to walk dependently. Also, the cervical devices and subaxial alignment were stable.

NR indicates not reported; Values are *n*.

^a^ Halo vests were used only to protect the spinal fusion in those cases with non-instrumented fusions and a better fusion rate with instrumented fusion was found.

The biggest threat to children with MPS may be damage to the heart and respiratory system, which also means great risk of anesthesia. Therefore, adequate preoperative preparation, strict intraoperative monitoring, and postoperative management are very important. Multidisciplinary consultations should be conducted before operation, including anesthesiology, intensive care medicine, pediatric cardiovascular medicine, pediatric respiratory medicine, metabolic and genetic diseases, and spinal surgeons. The assessment of cardiopulmonary function and risk of anesthesia are particularly important. The intensive care unit should be fully prepared before the operation. At the same time, electrophysiological detection equipment should be prepared for intraoperative monitoring, and 3D printed models should be prepared to facilitate surgeons' accurate understanding of complex structures during operation. Fine-cut 3D reconstructed preoperative studies should be carried out to make the most suitable choice among the different surgical options (7). Anesthesia should be performed by an experienced anesthesiologist, and fiberoptic intubation may be useful, considering the GAG-induced tracheal deformities. A right atrial central venous catheter is placed sometimes, as the risk of dangerous cardiac events is increased in patients with MPS (12). Intensive care should be obligatory, owing to the increased risk of pulmonary insufficiency (12).

In view of the rarity of the disease, the choice of surgery is also controversial. A study published in 2017 advocated that, for patients without craniocervical instability, stand-alone craniocervical decompression is feasible and osteosynthesis is not necessary (12). Decompression surgery without prophylactic osteosynthesis reduces procedure time, iatrogenic trauma, and hospital time. The overall mortality in their case series is lower than that in the applicable literature, but their rate of respiratory-related complication is higher (5). In the study of Krenzlin et al. (12), although the first stand-alone decompression surgery yielded good postoperative results, the reoperation rate was high (60% in type I, 40% in type IVA, and 50% in type VI).

Clinically relevant restenosis, which was believed to be caused by underlying MPS, was the main reason for readmission and re-surgery. GAG deposits in connective tissues and dura mater result in increased rigidity, counteracting the anatomical misalignment and anticipated hypermobility. This balance is the basis for stand-alone craniocervical decompression, but with the development of medical treatment in recent years, the medical treatment for the etiology of MPS can be carried out synchronously with the operation at an early age, the deposition of GAG is slowed down, and the structural damage caused by the surgery cannot be compensated quickly, which leads to increased potential risk of instability. Giussani et al. (7) believed that removal of the posterior ring of C1, hypertrophied ligamentum flavum, and occipitoatlantal membrane in posterior decompression surgery inevitably aggravates craniovertebral junction instability and may expose patients to acute post-traumatic myelopathy after even a minor trauma in flexion. Besides, in the study of stand-alone surgery, the average age of those 15 patients was approximately 15 years (i.e., they were old enough for reoperation). However, with the development of diagnostic technology, these patients are being diagnosed at a younger age (i.e., at ∼1 or 2 years), during which surgery is difficult and reoperation seems impossible. Therefore, it is important to perform early internal fixation to stabilize the spine and minimize the possibility of re-surgery.

Occipitocervical fusion is the most commonly used surgical strategy, because it is believed to be difficult to establish satisfactory stability in patients with ligamentous laxity if only C1-C2 fusion is performed (9, 13–15). Besides, as surgical intervention is recommended at an early age in such cases, the small size of these young patients is the main challenge for spinal surgeons when establishing stability *via* C1-C2 fusion alone because there is no dedicated cervical pediatric instrumentation available and even the smallest one for an adult is too big compared with the smaller vertebral dimensions. This makes it more difficult to put the screws into the correct position, especially for those with developmental deformity of the cervical vertebra (9, 14). However, in our center, we still prefer C1-C2 fusion because we try to preserve the child's cervical spine flexion-extension capacity as much as possible. Reducing unnecessary disabilities are of great importance in improving the children's quality of life and integrating them into society. Besides, C1-C2 fusion can also make the surgery possible for patients who cannot afford expensive treatments. Based on our experience and study of the literature, C1-C2 fusion is feasible and appropriate even for young children, especially with the help of new technologies such as 3D printing and intraoperative 3D image-based navigation system. Our center has successfully performed several cases of C1-C2 fusion operations for children with MPS type IVA, and all of them have achieved satisfactory prognosis. The youngest patient was only 2 years old, and the clinical symptoms associated with cervical spinal cord compression improved remarkably after the operation; however, other problems occurred in the thoracolumbar segment a few months later.

Posterior cervical C1-C2 fusion is an important, yet difficult and risky, procedure in spinal surgery, and effective internal fixation techniques reduce the risk of the operation and improve the fusion rate. In 1910, Mixter and Osgood (16) first reported the technique of C1-C2 stabilization by tying the odontoid with silk to treat a chronic non-healing odontoid fracture. Although the silk was replaced by wires and various modifications to the technique have been made in subsequent years, the disadvantages of the sublaminar wire technology are obvious: first, the spinal cord can be easily be injured during the passing of two separate sublaminar wires under both C1 and C2 laminae (17); second, the fixation offers poor rotational stability; and third, the early micro-movement reduces the fusion rate. Besides, the C1 posterior arch and C2 lamina/spinous processes should be intact (17). The use of interlaminar clamps and transarticular screw fixation of C1-C2 were first introduced in 1975 and 1979, which improved biomechanical stability and fusion rate (18–21). Goel and Laheri (22) used C1 lateral mass–C2 pars screw construct connected by posterior cervical plates to achieve posterior fixation, and Harms and Melcher (23) then modified the Goel technique. They used C1 lateral mass screws and C2 pedicle screws connected by rods to achieve rigid fixation, which provided great stability and fixation rate and reduced injury to the nerve root and vertebral artery. The Harms technique has been widely accepted by spine surgeons worldwide and is considered the gold standard (24, 25). For patients with MPS, the rate of structural variation of the cervical vertebrae is very high, and the standard Harms technique may not be achieved; thus, some alternatives are needed. The C1 “pedicle” screw fixation technique provides higher pull-out strength and avoidance of the neurovascular elements, but the risk of injury to the vertebral artery is higher than that of C1 lateral mass screws, and fracture may occur in the posterior arch. The C1 posterior arch crossing screw technique can be used as an alternative for failed conventional atlantoaxial screw placement or failed screw placement. C2 intralaminar screw fixation provided a salvage technique in cases of failed C2 pedicle screw placement or instances of high-riding vertebral artery (26, 27). Because of considerable anatomic variability, none of the previously described techniques allow absolute safety, and there are some hybrid constructs of those techniques to address complex clinical situations (17).

Cervical pedicle screws (CPSs) and rods offer greater stability than other techniques, but the risk of serious complications, such as injury to the vertebral artery, spinal cord, and nerve roots, remains. Computer-assisted surgery serves as an effective tool in improving accuracy. Preoperative and postoperative CT data do not match because they were obtained at different positions. Intraoperative 3D image-based navigation can reduce the discrepancy and facilitate safe and accurate insertion (28–32). The O-arm is an intraoperation image system that allows high-definition 3D navigation and thus facilitates a more accurate, convenient, and quick insertion of the screws. The O-arm-based navigation can reduce CPS malposition but cannot completely prevent it. As the position of the cervical structure can easily change, especially in young patients whose cervical structure is very small, the discrepancy of alignment between 3D image and CPS insertion reduces the accuracy. In our practice, we use the O-arm-based navigation system to determine insertion points and explore insertion paths using a micro-grinding drill. According to our practice, 3D printing may make this difficult and dangerous operation easier and safer, especially for patients with severe deformities that make placement of screws difficult.

**Figure 3:**
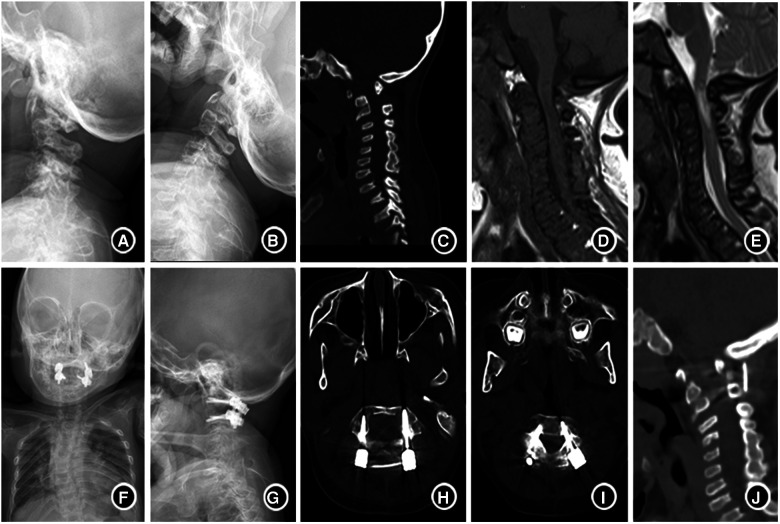
A 4-year-old boy with type IVA mucopolysaccharide disease (**A,B**). Lateral plain-film flexion-extension studies before the surgery showed uneven cervical bone density, flattened vertebra, atlantoaxial instability. (**C**) CT suggested atlantoaxial subluxation and odontoid process absence; (**D,E**) MRI indicated spinal canal stenosis, high cervical spinal cord compression and edema; (**F,G**) showed that the anatomical position of atlantoaxial returned to normal half a year after operation. (**H–J**). CT showed that the screw position was good, and the bone graft was fused with the atlantoaxial vertebra 6 months after operation.

With the in-depth understanding of MPS, advances in related monitoring tools, and progress in anesthetic techniques, the surgery for MPS has become safer than ever; thus, we advocate early decompression, posterior fixation and fusion of C1-C2 with CPS, and bone graft for children with MPS. Based on the experience of our center, we believe this is the most beneficial surgical management patients with MPS with for cervical involvement. (Typical case is presented in Figure 3).

Two main types of bone grafts are available for fusion: autogenous and allogenous. Allograft technologies were most commonly used to eliminate donor-site morbidity and complications related to autogenous bone graft. A study in 2017 compared structural allograft and autograft for instrumented atlantoaxial fusions in a series of 32 pediatric patients (33). In this study, the outcomes and fusion rates were similar regardless of whether an autograft or allograft was used; fusion time was increased when using allograft technologies, but blood loss was decreased and donor-site morbidity was avoided. However, as more clinical cases were studied and further long-term follow-up were conducted, fusion failure when using allograft has been observed. As reoperation of pediatric patients with failed fusion can be challenging, we believe that autograft should be the first priority to increase the success rate as much as possible. Available autograft bones include the iliac crest, ribs, and external plate of the skull. The anatomical features of the skull make it a good site for harvesting autogenous bones, which reduces injury to other sites. For C1-C2 fusion, the external plate of the skull should be the first choice. However, the skull plate cannot be used when occipitocervical fusion is needed, as the skull cannot be nailed without the outer plate. In a young child, the iliac crest is very thin and cartilage-based, which limits its application, and harvesting of the ribs can do a lot of damage.

In addition, osteoporosis is a common condition in patients with MPS. The term “dysostosis multiplex” is used to describe the abnormalities of MPS diseases. Osteoporosis has also been described in animal models of MPS. GAGs accumulate in all cells related to bone formation and remodeling in animal models of MPS, interfering with the normal formation of mineralized cartilage septa, which is required for osteoblasts and osteoclasts in the formation of new bones. Besides, the risk of poor bone mineralization of patients with MPS increases with malnutrition and reduction of physical activities caused by pain or exercise intolerance (34, 35). The pathophysiological basis of osteoporosis in patients with MPS is not completely understood. Considering the possibility of osteoporosis and the smaller bone structure of young patients with MPS, external fixation with brace for 3 to 6 months is needed to reduce internal fixation failure and thus avoid reoperation. Anti-osteoporosis therapy may be effective for postoperative recovery.

## Conclusions

The compression of the cervical spinal cord due to various reasons is the main life-threatening factor in children with MPS. Advances in imaging technology, especially MRI, enable us to detect abnormalities of the spine and spinal cord as soon as possible and perform surgical intervention before neurological deterioration and loss of function. Although the number of cases is limited, decompression, autogenous bone fusion, and internal fixation with screws seem to be the best treatment options for children with MPS at present. With the help of various preoperative high-resolution reconstruction techniques, intraoperative 3D image-based navigation system, and 3D printing technology, C1-C2 fixation is feasible and safe in most cases, which preserves the flexion-extension capacity of the cervical spine as much as possible. In view of the high incidence of spinal diseases in children with MPS, we recommend that once MPS is diagnosed, relevant tests should be carried out as soon as possible to rule out cervical vertebra–related diseases.
